# Efficacy of Fowlpox Virus Vector Vaccine Expressing VP2 and Chicken Interleukin-18 in the Protection against Infectious Bursal Disease Virus

**DOI:** 10.3390/vaccines11111716

**Published:** 2023-11-14

**Authors:** Ibrahim Eldaghayes, Lisa Rothwell, Michael Skinner, Abdunaser Dayhum, Pete Kaiser

**Affiliations:** 1 Institute for Animal Health, Compton, Berkshire RG20 7NN, UK; 2 Department of Microbiology and Parasitology, Faculty of Veterinary Medicine, University of Tripoli, Tripoli P.O. Box 13662, Libya; 3 The Roslin Institute and R(D)SVS, University of Edinburgh, Easter Bush, Midlothian EH25 9RG, UK; 4 Section of Virology, Department of Medicine, St Mary’s Campus, Imperial College London, Norfolk Place, London W2 1PG, UK; 5 Department of Preventive Medicine, Faculty of Veterinary Medicine, University of Tripoli, Tripoli P.O. Box 13662, Libya

**Keywords:** chicken interleukin-18, vaccine adjuvant, recombinant Fowlpox virus FP9, fpIBD1

## Abstract

In mammals, the role of interleukin-18 (IL-18) in the immune response is to drive inflammatory and, normally therefore, anti-viral responses. IL-18 also shows promise as a vaccine adjuvant in mammals. Chicken IL-18 (chIL-18) has been cloned. The aim of this study was to investigate the potential of chIL-18 to act as a vaccine adjuvant in the context of a live recombinant Fowlpox virus vaccine (fpIBD1) against Infectious bursal disease virus (IBDV). fpIBD1 protects against mortality, but not against damage to the bursa of Fabricius caused by IBDV infection. The Fowlpox virus genome itself contains several candidate immunomodulatory genes, including potential IL-18 binding proteins (IL-18bp). We knocked out (Δ) the potential IL-18bp genes in fpIBD1 and inserted (::) the cDNA encoding chIL-18 into fpIBD1 in the non-essential ORF030, generating five new viral constructs –fpIBD1::chIL-18, fpIBD1ΔORF073, fpIBD1ΔORF073::chIL-18, fpIBD1ΔORF214, and fpIBD1ΔORF214::chIL-18. The subsequent protection from challenge with virulent IBDV, as measured by viral load and bursal damage, given by these altered fpIBD1 strains, was compared to that given by the original fpIBD1. Complete protection was provided following challenge with IBDV in chicken groups vaccinated with either fpIBDIΔ073::IL-18 or fpIBD1Δ214::IL-18, as no bursal damage nor IBDV was detected in the bursae of the birds. The results show that chIL-18 can act as an effective vaccine adjuvant by improving the fpIBD1 vaccine and providing complete protection against IBDV challenge.

## 1. Introduction

Infectious bursal disease (IBD) was first recognized in Gumboro, Delaware, USA, in 1962 [1], hence its alternative name, Gumboro disease. IBD is known to cause high economic losses for the poultry industry throughout the world because of its high morbidity and mortality rates in infected chickens [2]. Before the 1980s, IBD was well controlled by vaccination; however, by the end of the 1980s, many vaccination failures were reported worldwide, due to the emergence of variants and, later on, very virulent strains of the IBD virus (IBDV). The new strains of IBDV are called “variant” strains in the USA [3], and “hypervirulent” or “very virulent” IBDV (vvIBDV) strains in Europe [4].

Recombinant Fowlpox viruses have been used as a vector to express targeted genes from many avian pathogens, such as Newcastle disease virus [5,6], Avian influenza virus [7], Turkey rhinotracheitis virus [8], Marek’s disease virus [9], and IBDV [10].

IBDV is a Birnavirus, characterized by having a bi-segmented double-stranded RNA genome [2]. IBDV encodes five proteins of which VP2 is the capsid protein. VP2 is known as the host-protective antigen [11]; hence, it has been selected to produce new recombinant vaccines. VP2 was expressed in the FP9 strain of the Fowlpox virus [12] to generate a recombinant vaccine, fpIBD1 [10]. In a previous study, fpIBD1 was able to provide significant levels of protection against the challenge from IBDV. However, this protection was mainly against mortality, but not against damage that occurred by IBDV to the bursa of Fabricius [10]. In addition, the protection of the fpIBD1 vaccine was dependent on other factors such as the titer of IBDV and also the difference in the major histocompatibility complex (MHC) haplotype of the specific pathogen-free (SPF) chickens used in the experiment [13].

Interleukin-18 (IL-18) is well known as a pro-inflammatory cytokine, as well as an interferon-gamma-inducing factor (IGIF), and plays an important role in the development of T-helper type 1 (Th1) cells, which drive cell-mediated immune responses. As IL-18 is an inducer for the Th1 response, it therefore seemed logical to investigate the efficacy of IL-18 as a vaccine adjuvant. Chicken IL-18 (chIL-18), originally identified in an EST database, was cloned and expressed [14].

ChIL-18 is known to enhance the immune responses and protection efficacy of vaccines against avian diseases, e.g., Newcastle disease [15], infectious bronchitis [16], avian influenza H9N2 [17], infectious laryngotracheitis [18], and chicken coccidiosis [19].

It was claimed that ORF FPV073 (in the Fowlpox virus genome) was a homologue of human IL-18 binding proteins (IL-18bp) and an orthologue of IL-18bps from Molluscum contagiosum virus, Swinepox virus, and Vaccinia virus [20]. It was later shown that ORF FPV214 is more likely to be the correct assignment [12]. Thus FPV214, but not FPV073, aligns with a conserved motif (97YWxxxxxFIEHL108 in humans) in the other IL-18bps. In contrast, FPV073 contains a GxGxxG nucleotide-binding motif and shows the highest similarity to a tyrosine-protein kinase. It seemed logical to delete IL-18bp from the vector containing chIL-18 as an adjuvant. Therefore, both Fowlpox virus ORFs 073 and 214 were knocked out, separately, from fpIBD1. ChIL-18 was then inserted into the non-essential [21] PC-1 gene (ORF FPV030) of fpIBD1. Then, the protection provided by these new vaccines was compared to the original fpIBD1, in terms of clinical signs, bursal damage, and viral loads, in the bursa of Fabricius following challenge with virulent IBDV.

## 2. Materials and Methods

### 2.1. Chickens

Outbred Rhode Island Red (RIR) chickens were used in all animal experiments. The chickens were bred at the Institute for Animal Health (IAH), were free from specific pathogens including IBDV, free of maternal IBDV antibodies, and maintained in isolation in wire-floored cages with free access to feed and water. All animal experiments were carried out in the experimental animal house (EAH) at the IAH. All procedures were carried out according to UK Home Office guidelines for animal welfare.

### 2.2. Viruses

**Figure 1:**
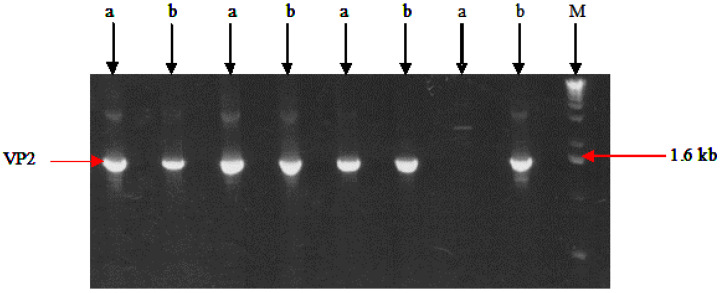
PCR products from fpIBD1 showing the VP2-specific band (~1.6 kb). Lanes (a) Primers VP2 F (+BamHI site) and VP2 R (+BamHI site). Lane (b) Primers VP2 F (+EcoRI site) and VP2 R (+NotI site). M—DNA marker.

**Figure 2:**
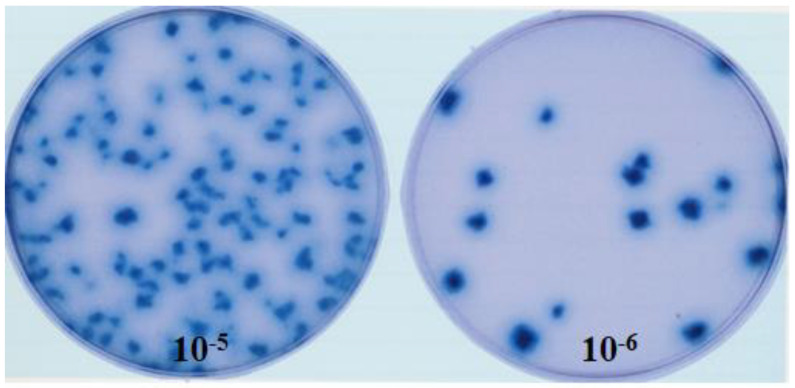
Blue plaques of fpIBD1 on X-gal overlay Agar (two different dilutions).

fpIBD1 is a recombinant vaccine originating from the Fowlpox virus FP9 [10]. fpIBD1 expresses the VP2 protein of the IBDV F52/70, which has been inserted in the BglII insertion site in the ORF FPV002 as a β-galactosidase fusion protein under the control of the early/late promoter of the Vaccinia virus p7.5 (Figure 1). Chicken embryo fibroblasts (CEFs) were prepared from 9 to 10-day-old embryonated eggs. CEF cells were provided in either Petri dishes or different sizes of T flasks; fpIBD1 was grown on CEF with the presence of growth media of 1X 199 medium (Sigma-Aldrich^®^ Brand, Merck KGaA, Darmstadt, Germany) at 37 °C in 5% CO_2_ (Figure 2).

The vIBDV strain F52/70 [22] was used, and its titer was kindly determined by Dr Adriaan van Loon (Intervet International BV (MSD AH), Boxmeer, The Netherlands) [23]. Based on earlier studies, the dose of virus selected was 10^2.3^ EID_50_ vIBDV strain F52/70, which can overcome the protection provided by fpIBD1 and cause damage to the bursa of Fabricius, which can be measured as bursal lesion score in 2–3-week-old RIR chicks (Davison TF, personal communication).

### 2.3. Generation of Novel fpIBD1 Recombinants

**Figure 3:**
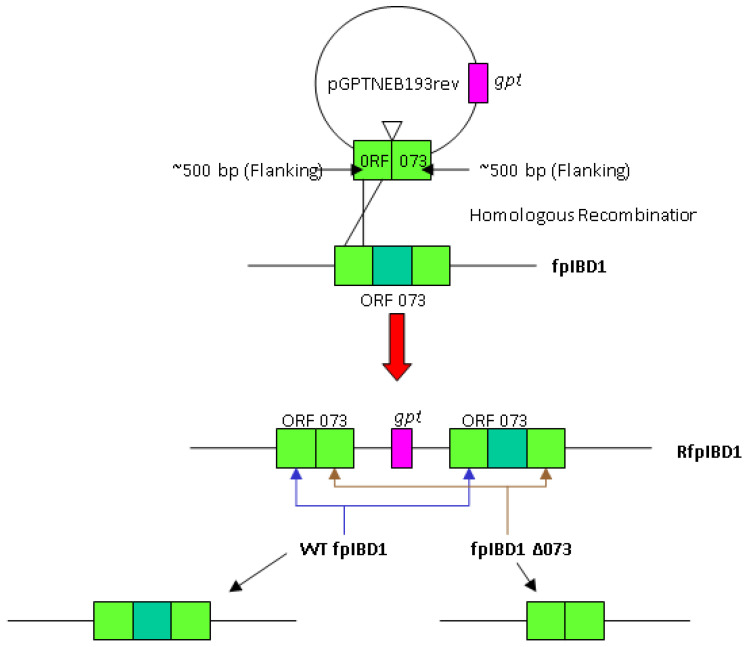
Homologous recombination between flanking sequence of ORF073 encoded by the plasmid and those in fpIBD1. The resulting recombinant fpIBD1 (RfpIBD1) was selected as gpt+. The selection of gpt− virus may result in either wild-type fpIBD1(WT fpIBD1) or knockout 073 (fpIBD1Δ073). The same method was used to generate knockout ORF214.

fpIBD1 mutants with knockout of IL-18bp genes were selected using trans-dominant selection [24] for the *Escherichia coli* guanine phosphoribosyltransferase (gpt) gene and checked by PCR [21]. Deleted recombination constructs, containing 50 bp from either end of Fowlpox virus ORFs 073 and 214, as well as 500 bp flanking sequences (Figure 3), were inserted into the BamHI and HindIII restriction sites of vector pGNR [21]. The constructs were then transfected, using Lipofectin (Invitrogen, Waltham, MA, USA), into CEF infected with fpIBD1. Following 37 °C in 5% CO_2_ overnight incubation, the culture medium was discarded and replaced with 1X 199 medium + 2% newborn bovine serum (NBBS) containing mycophenolic acid, xanthine, and hypoxanthine (MXH) solution and re-incubated for 2–4 days until a cytopathic effect was apparent. The virus was released by freeze/thawing the culture three times, and then plaqued under MXH selection.

Gpt+ recombinant clones were plaque-purified three times in MXH selective medium and then further purified without selection until they became gpt− (as determined by failure to plaque under MXH selective medium), which was confirmed by PCR.

For the production of recombinants expressing chIL-18, a recombination vector was constructed. Briefly, pGEM-T::chIL-18 was digested with NotI, and chIL-18 was then inserted into pEFgpt12S (containing S promoter). Sp+chIL-18 was then inserted into the PC-1 plasmid (pFPV-PC-1) to result in pFPV-PC-1::Sp+chIL-18, which was the target clone in which the chIL-18 gene, under the control of a synthetic early/late promoter, was inserted into the non-essential PC-1 gene (ORF FPV030) in a plasmid carrying the gpt gene under the control of the Vaccinia virus p7.5 promoter. CEF infected with parental fpIBD1, fpIBD1Δ073, and fpIBD1Δ214 viruses were then transfected with the pFPV-PC-1::S-promoter/chIL-18 plasmid. Gpt+ recombinants were selected and plaque-purified three times. Insertion of the expression and selection cassette into the PC-1 gene was confirmed by PCR. The six different recombinant viruses were titrated. Viruses carrying the gpt reporter gene (i.e., those that contain chIL-18) always had lower titers (Table 1).

### 2.4. Cloning Strategy

**Figure 4:**
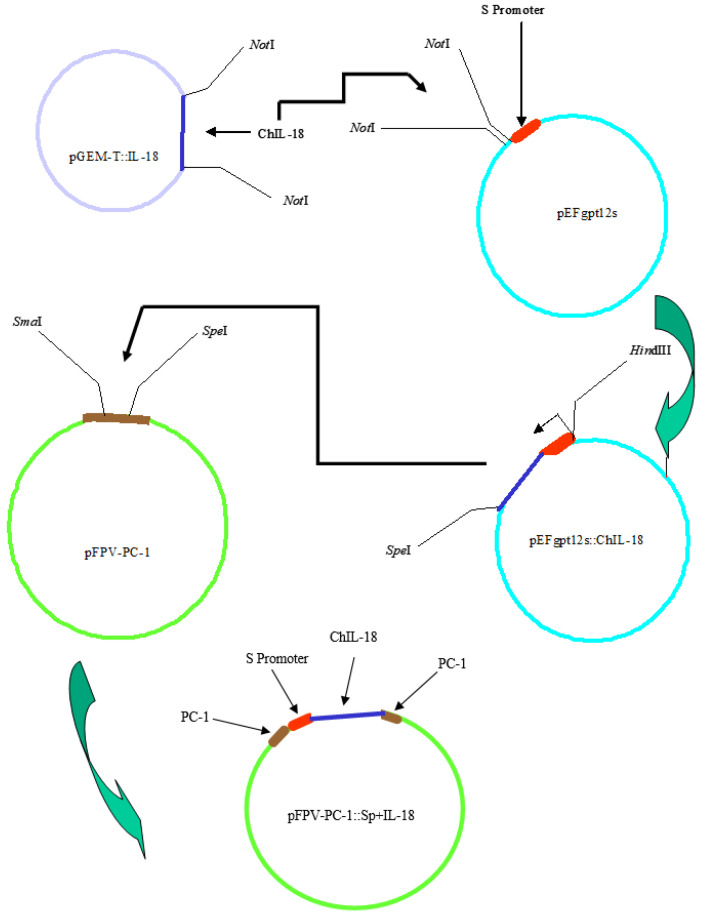
Cloning strategy resulting in the target plasmid containing the S promoter, ChIL-18, and flanking sequences of the PC-1 gene.

Figure 4 shows the cloning strategy to generate the plasmid pFPV-PC-1::Sp+ChIL-18. Briefly, pGEM-T::ChIL-18 was digested with *Not*I, and chIL-18 was then inserted into pEFgpt12S (containing the S promoter). This cassette (Sp+ChIL-18) was then excised with *Hin*dIII and inserted into the PC-1 plasmid (pFPV-PC-1) to result in pFPV-PC-1::Sp+ChIL-18, which was the target clone to insert ChIL-18 into the PC-1 gene of fpIBD1 and altered fpIBD1 strains.

### 2.5. Experimental Design

A total of 70 day-of-hatch chicks were divided into 14 groups; 7 groups of 7 birds per group were challenged with IBDV, with 7 groups of 3 birds per group as a control, in 4 separate rooms (Table 2).

Chicks (groups 1 and 2) were used as unvaccinated control groups; the first group as a negative control without challenge, and the second group was a positive control as it was challenged with the IBDV. Chicks (groups 3–14) received the first vaccination of 50 μL volume at one week of age with 10^7^ pfu fpIBD1 and altered fpIBD1. The vaccination site was, as per Fowlpox vaccination, in the wing-web, with the site of vaccination punctured many times using a hypodermic needle, and a booster vaccination with the same doses was carried out two weeks later. Ten days following the booster vaccination, chickens in groups from 2 to 8 were challenged with IBDV. The challenge dose of 10^2.3^ EID_50_ of IBDV strain F52/70 in a volume of 100 μL was given by intranasal route. Birds from the challenged unvaccinated group (group 2) were bled daily after the day of challenge. Blood (50 μL) was taken into a 350 μL RTL buffer, after puncturing the wing vein with a needle. Five days after challenge, all birds in all groups were killed and the bursae removed and cut into 3 pieces. A small piece was taken for RNA extraction, and 2 larger pieces were taken, one for immunohistochemistry and the other one into formalin solution for H&E staining to determine bursal damage.

### 2.6. Sample Processing

#### 2.6.1. RNA Extraction

Bursal tissue (~30 mg) was homogenized using a Bead mill (Retsch MM300, RETSCH GmbH, Haan, Germany). Briefly, the bursal tissue was placed in a 2 mL Safe-lock Eppendorf tube with 600 μL lysis buffer RLT. A 5 mm stainless steel bead was added per tube. The tubes were placed in adaptors for the bead mill and run for 4 min at 20 Hz. RNA was extracted from tissues and blood using the RNeasy mini kit (QIAGEN Ltd.—UK, Manchester, UK) as per manufacturer’s instructions and stored at −70 °C.

#### 2.6.2. Frozen Sections for Immunohistochemical Staining

Each bursal tissue sample was put on a 2.5 cm^2^ cork tile. The bursal tissue sample was then covered with Tissue-Tek^®^ OCT^TM^ Compound and snap-frozen in a dry-ice/iso-pentane bath and then transferred immediately to a tank of liquid nitrogen. Frozen blocks were then stored at −70 °C and sections were prepared for immunohistochemical staining.

Bursal tissue sections of 6–8 μm for each section were cut using a cryostat. Each section was picked up and placed on a glass slide and fixed in acetone for 10 min and left to air-dry. Staining of bursal tissue sections was performed using a Vectastain^®^ ABC α-mouse IgG HPR staining kit (Vector Laboratories, Burlingame, CA, USA) as per the manufacturer’s protocol. Image-Pro^®^ Plus software version 4.0 was used to count the number of positively stained cells as a percentage of the field of view at a magnification power of 100×. The monoclonal antibodies used were R63 [3] for IBDV and AV20 [25] for B cells.

#### 2.6.3. H&E Staining

Each bursal section from every bird was put into 40 mL formalin and stained with hematoxylin and eosin (H&E) to look for bursal damage. Briefly, the bursal tissue samples fixed with 4% paraformaldehyde were dehydrated in a graded series of ethanol, embedded in paraffin, and sectioned serially at a thickness of 4 μm. The obtained tissue sections were collected on slides and stained with H&E.

### 2.7. Real-Time Quantitative RT-PCR

Quantitative real-time RT-PCR was carried out for IBDV and 28S [26]. A probe against 28S rRNA was used to control for variations in quantities of total RNA between samples. IBDV-specific forward and reverse primers were used targeting IBDV segment A [27]. Primers and probes used are shown in Table 3. All probes were labeled with the fluorescent reporter dye 5-carboxyfluoroscein (FAM) at the 5′ end and the quencher dye N,N,N,N′-tetramethyl-6-carboxyrhodamine (TAMRA) at the 3′ end (PE Applied Biosystems).

Reagents from the TaqMan^®^ EZ RT-PCR kit (PE Applied Biosystems, Waltham, MA, USA) were used for the RT-PCR. RNA is first reverse-transcribed to cDNA by r*Tth* DNA polymerase (not shown) and amplified by PCR. Separation of the reporter dye from the quencher dye leads to an increase in the fluorescence during each extension cycle of the PCR. Amplification and detection of specific products were performed using the ABI PRISM^TM^ 7700 Sequence Detection System, with cycle profile as follows: 1 cycle of 50 °C for 2 min, 96 °C for 5 min, 60 °C for 30 min, and 95 °C for 5 min, and 40 cycles of 94 °C for 20 s and 59 °C for 1 min. Results are expressed in terms of the cycle threshold (C_t_), a statistically significant increase in fluorescence.

### 2.8. Construction of Standard Curves for Quantitative PCR and RT-PCR Assays

In every experiment, 28S rRNA standard curves were generated and included. For the generation of standard curves, a regression analysis of the mean values for the log_10_ diluted RNA was used. The slope of the regression line for each of the target RNAs gives a measure of the efficiency of the reaction for that particular prime/probe combination. To control for the variation in input test RNA, due to variations between samples and in RNA preparation, the C_t_ values for each sample were standardized using the C_t_ value of the 28S rRNA product for the same sample. All 28S rRNA C_t_ values were similar for samples within and between experiments. The efficiency of the 28S rRNA reaction, which is seen in the slope of the log_10_ dilution series regression line, was used to calculate differences in input total RNA.

**Figure 5:**
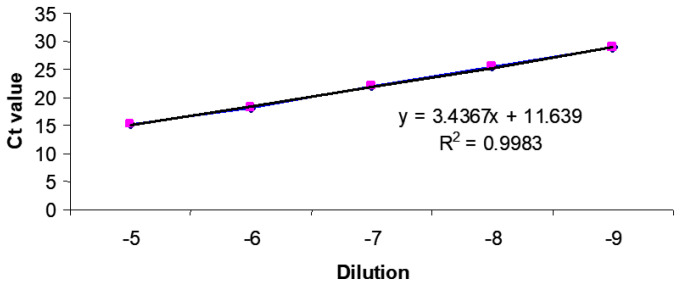
Real-time quantitative PCR: (A) Standard curve for pCI-neo::VP2.

Standard curves for the DNA-specific reactions were generated from real-time PCR reactions on serial log_10_ dilutions of DNA standards (usually plasmid DNA containing the same DNA template), and were included in every experiment. Dilutions of 10^−1^ to 10^−9^ were prepared in DEPC-H_2_O. Regression analysis of the mean values for the log_10_ diluted DNA plasmid was used to generate a standard curve (Figure 5).

### 2.9. Statistical Analysis

Data variations between the six vaccinated groups were analyzed by one-way ANOVA. Then Duncan’s Multiple Range test (DMRT) (a post hoc test) was used to measure specific differences between pairs of means by using IBM SPSS version 24. *p* values less than 0.05 were considered statistically significant in all statistical analyses.

## 3. Results

### 3.1. Confirmation of fpIBD1::1L-18, fpIBD1Δ073::IL-18 and fpIBD1Δ214::IL-18

**Figure 6:**
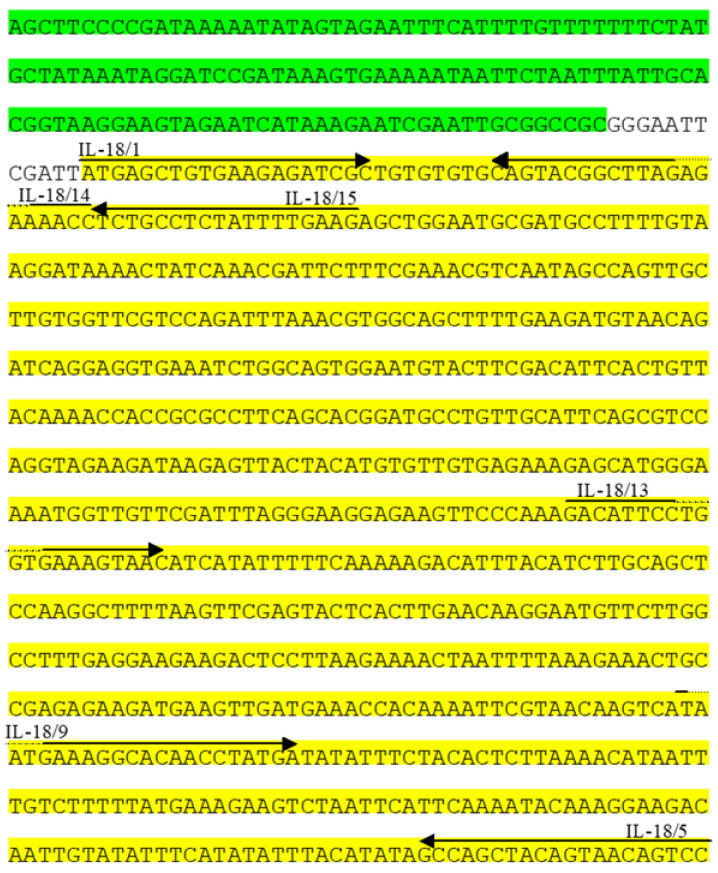
Sequencing of the insert. S promoter sequences are shown with a green background and ChIL-18 sequences with yellow. The ChIL-18 primers used in this study and their positions are shown. The sequence in black is the remaining sequence between the NotI site and the insert (ChIL-18) in the original vector pGEM-T Easy.

**Figure 7:**
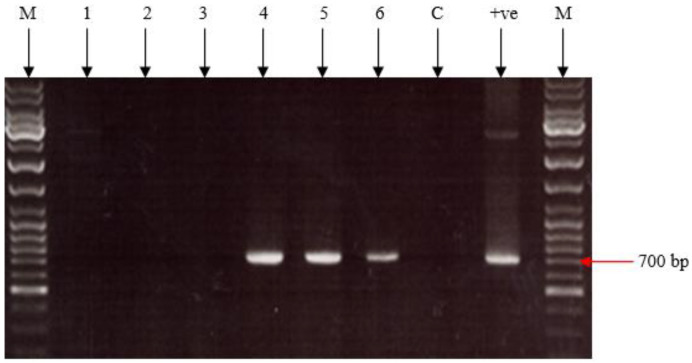
PCR products from DNA preps of fpIBD1 (1), fpIBD1Δ073 (2), fpIBD1Δ214 (3), fpIBD1::IL-18 (4), fpIBD1Δ073::IL-18 (5), and fpIBD1Δ214::IL-18 (6), using primers IL-18/1 F and IL-18/5 R. (+ve): pGEM-T Easy::IL-18. (C): negative control. (M): DNA marker. The band in lane 6 is less intense due to a lower DNA concentration being used as a template in the PCR.

Growth after three passages in media containing MXH, for gpt+ selection, indicated that the plasmid was inserted into the PC-1 gene. To confirm this, CEF in T25 flasks (containing 5 mL 1X 199 medium + 2% NBBS + MXH) infected with 100 μL of each virus was incubated at 37 °C in 5% CO_2_ for 4–6 days. The resulting CPE was clear and the viruses were then freeze/thawed three times. DNA preps for all six viral vaccines were prepared. PCR amplifications were set up using primers IL-18/1 F and IL-18/5 R (Table 3, Figure 6). ChIL-18 was inserted into fpIBD1 (fpIBD1::1L-18) and altered fpIBD1 (fpIBD1Δ073::IL-18 and fpIBD1Δ214::IL-18) strains successfully (Figure 7).

**Figure 8:**
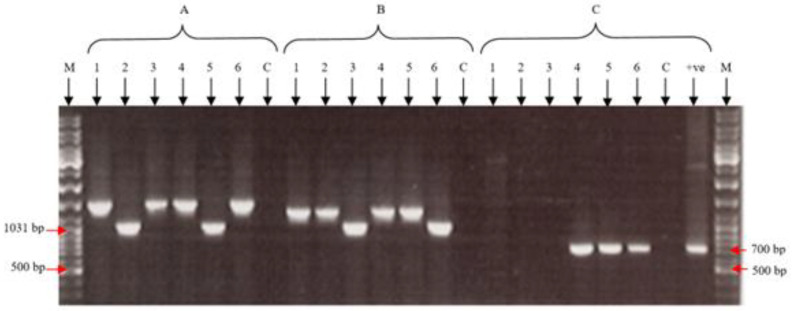
PCR products from DNA preps of fpIBD1 (1), fpIBD1Δ073 (2), fpIBD1Δ214 (3), fpIBD1::IL-18 (4), fpIBD1Δ073::IL-18 (5), and fpIBD1Δ214::IL-18 (6), using (A) primers 73Flk F and 73Flk R, (B) primers 214Flk F and 214FlkR and (C) primers IL-18/1 F and IL-18/5 R. +ve—pGEM-T Easy::IL-18. C—negative control. M—DNA marker.

After eight rounds of blue plaque purification, all six viral vaccines were cultured in CEF cells. Insertion and expression of chicken IL-18 into the FPV genome were confirmed using PCR on DNA preps from all viral vaccines (Figure 8), DNA sequencing performed on the RT-PCR products (Figure 6), and an indirect immunofluorescence assay.

### 3.2. Protection from IBDV Challenge by the Recombinant Vaccine fpIBD1 and the New Viral Constructs

**Figure 9:**
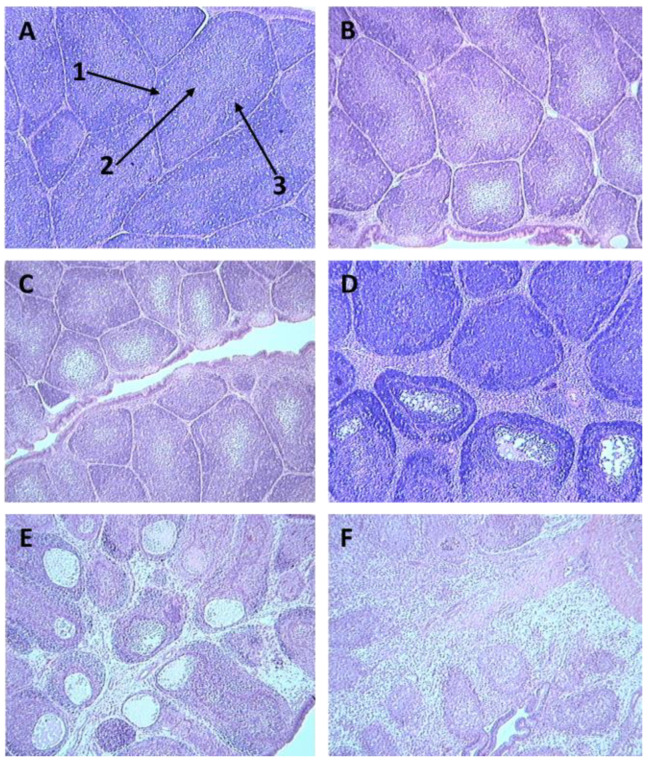
Sections of bursae stained with H&E from birds infected with vIBDV strain F52/70, showing different bursal damage scores as detailed in Table 3: (**A**) = bursal damage score of 0, (**B**) = bursal damage score of 1, (**C**) = bursal damage score of 2, (**D**) = bursal damage score of 3, (**E**) = bursal damage score of 4, (**F**) = bursal damage score of 5. Arrows indicate the cortex (1), medulla (2), and cortico-medullary junction (3) of a bursal follicle.

Within 2 days, birds vaccinated with parental fpIBD1 and altered fpIBD1 developed pocks, at the site of inoculation, which disappeared by 10 days. Protection from IBDV challenge by fpIBD1 recombinant vaccine and the altered fpIBD1 was measured by the appearance of IBD clinical signs, the bursal damage scoring index of Muskett et al. [28] (Table 4 and Figure 9), and viral loads (IBDV) in the bursa, using immunohistochemical staining and real-time quantitative RT-PCR, at 5 days post-infection (dpi).

Severe bursal damage was observed for all infected, unvaccinated birds (Table 4). Four out of five (80%) fpIBD1-vaccinated birds were not protected. Three birds out of five (60%) vaccinated with fpIBD1Δ073 were not protected. Only one bird out of five (20%) vaccinated with fpIBD1Δ214 was not protected.

**Figure 10:**
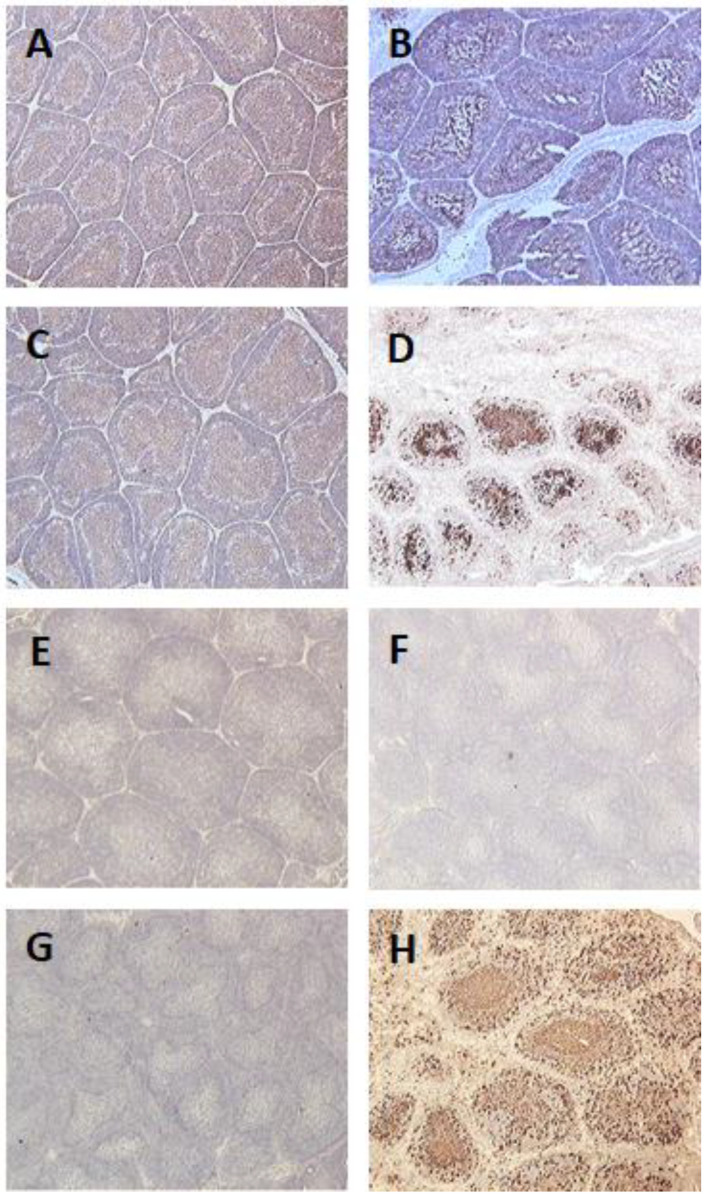
Bursal tissue sections from different groups taken at 5 dpi with the vIBDV strain F52/70: (**A**–**D**): The sections were stained with the anti-B cell monoclonal antibody, AV20, and counterstained with hematoxylin. (**A**): unvaccinated and unchallenged (-ve control), (**B**): vaccinated with fpIBD1 and challenged, (**C**): vaccinated with fpIBD1Δ214::IL-18 and challenged, (**D**): unvaccinated and challenged (+ve control). (**E**–**H**): Bursal tissue sections stained with the anti-IBDV monoclonal antibody, R63, and counterstained with hematoxylin at 5 dpi. (**E**): unvaccinated and unchallenged, (**F**): vaccinated with fpIBD1 and challenged, (**G**): vaccinated with fpIBD1Δ214::IL-18 and challenged, (**H**): unvaccinated and challenged. Challenge was with 10^2.3^ EID_50_ vIBDV strain F52/70 (100× magnification).

Interestingly, no bursal damage was seen for all birds vaccinated with viral constructs containing IL-18. In unvaccinated challenged birds, as IBDV targeted B cells in the bursae of infected birds, a huge depletion of B cells was obvious. Some B cell depletion was seen in the bursae of birds vaccinated with fpIBD1. Very little B cell depletion was seen in the bursae of birds that were vaccinated with the knockout viruses 073 and 214, and no B cell depletion was observed in the bursae of birds that were vaccinated with the viruses that contained chIL-18 (*p* = 0.002) (Figure 10).

### 3.3. Detection of IBDV Using Immunohistochemistry

Bursa tissues of unvaccinated and challenged chickens were swamped with the virus. Clear destruction of the bursa of Fabricius can be seen. On the other hand, no IBDV was detected in the bursa samples of vaccinated chickens (Figure 10).

### 3.4. Detection of IBDV Using Real-Time Quantitative RT-PCR

**Figure 11:**
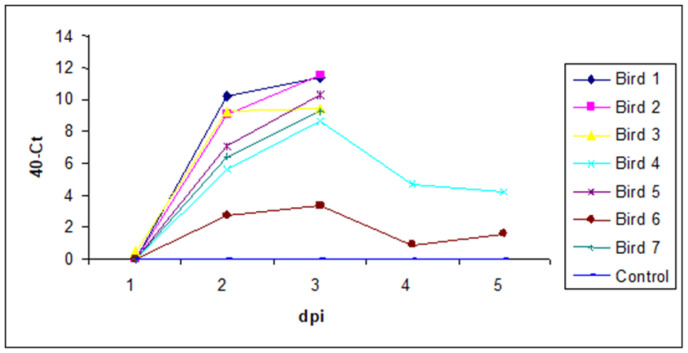
IBDV mRNA levels in the blood of unvaccinated birds (-ve control) and birds challenged with IBDV 10^2.3^ EID_50_ strain F52/70 were quantified by real-time quantitative RT-PCR. Birds nos. 4 and 6 survived until the end of the experiment, whereas the other birds reached the clinical end point by 3 dpi. Results are expressed as 40-Ct values.

Viral RNA in 50 μL of whole blood extracted from infected chickens (unvaccinated) over the first 5 dpi with vIBDV strain F52/70 (every 24 h) was quantified. The total number of birds in this group was seven birds. Five birds came to the clinical end-point by 3 dpi and two birds survived until the end of the experiment (5 dpi). The results show that IBDV levels in the blood reach a peak at 3 dpi. The two surviving birds had lower levels of IBDV in the blood compared to the other birds (Figure 11).

**Figure 12:**
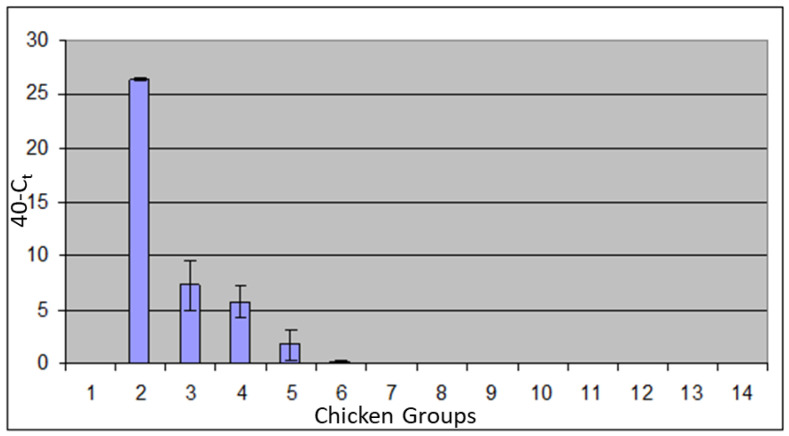
Viral loads (vIBDV strain F52/70) in the bursa 5 dpi: (1): -ve control; (2): +ve control (vIBDV); (3): fpIBD1; (4): fpIBD1Δ073; (5): fpIBD1Δ214; (6); fpIBD1::IL18; (7): fpIBD1Δ073::IL18; (8): fpIBD1Δ214::IL18; (9): fpIBD1; (10): fpIBD1Δ073; (11): fpIBD1Δ214; (12): fpIBD1::IL18; (13): fpIBD1Δ073::IL18; (14): fpIBD1Δ214::IL18; (Groups 2–8): Challenged with vIBDV; (Groups 9–14 and group 1): No challenge.

IBDV was detected in the bursa at very high levels in infected, unvaccinated birds (Figure 12). There was a low level of IBDV in the bursae of birds vaccinated with fpIBD1 or with fpIBD1Δ073 and lower levels still in the bursae of birds vaccinated with fpIBD1Δ214. Interestingly, hardly any IBDV was detected in the bursae of birds vaccinated with fpIBD1::IL-18, and even more interestingly, no virus was detected in the bursae of birds vaccinated with either fpIBDIΔ073::IL-18 or fpIBD1Δ214::IL-18 (Figure 12). The experiment was repeated and similar results were obtained.

## 4. Discussion

In chickens, it has been shown that the Th1/Th2 paradigm occurs in a similar way to that in mammals [29]. In order to have therapeutic efforts for a specific disease, it is important to first understand the protection that can be provided by the immune response and then apply the response to establish a strategy for vaccination. For example, to have a good immune response against viruses as an example of intracellular pathogens, priming immunization should be directed toward Th1 responses. This can be achieved by studying the cytokines that are produced during infection as well as what type of immune cell population has been affected. IL-18 has been known to induce interferon-gamma (IFN-γ) production and promote Th1 immunity; hence, it has been tested as an adjuvant in the fpIBD1 vaccine and proved to enhance immunity in vaccinated chickens against IBDV challenge.

Many cytokine profiles can now be easily measured during the course of any studied infection. This was the case for cytokine response and production in the bursa of Fabricius during IBDV infection [30]. Pro-inflammatory cytokine responses in the bursa of Fabricius were detected during IBDV infection by the increasing of the pro-inflammatory cytokines IL-1β, IL-6, and IL-8, and down-regulation of the anti-inflammatory cytokine TGF-β4. IFN-γ expression was increased in infected bursae, presumably reflecting this inflammatory response, which has been reported in earlier published results [31,32], suggesting cell-mediated immune responses are activated in order to overcome the infection. Chicken IFN-γ also has anti-viral activity [33], and the increased levels of IFN-γ mRNA are consistent with the enhanced production of IFN-γ proteins, which should have anti-viral activity. IL-18 is well known as IGIF and plays an important role in the development of Th1 cells, which drive cell-mediated immune responses. Cell-mediated immune responses are important in the protection of viral infections, and, therefore, it seemed logical to investigate the efficacy of IL-18 as a vaccine adjuvant against IBDV.

Pathogens continually evolve in order to evade the host’s immune response, and there is growing evidence that, in response to the intensive use of vaccines, some viruses have increased in virulence, overcoming vaccine-induced protection [34,35].

The requirements for an effective poultry vaccine include safety and stimulation of appropriate and long-lasting immune responses that protect against the pathogen and produce minimal side effects. New technologies such as recombinant viral vector vaccines may offer a solution and, indeed, experimental vaccines like these have provided encouraging results after challenge with IBDV [10]. New vaccine strategies have emerged in recent years that take advantage of recombinant DNA technology and improved immunological knowledge, for example, recombinant subunit vaccines that contain only the immunogenic part of a pathogen that could have many advantages over live attenuated or inactivated vaccines.

For expression in Fowlpox, the gene of interest, VP2 of IBDV in the fpIBD1, is placed next to a poxvirus promoter and inserted into the virus genome by homologous recombination or direct ligation. Using poxvirus promoters is necessary as the poxvirus transcriptional apparatus does not recognize cellular and other viral promoters.

fpIBD1 is a recombinant FPV, which expresses VP2 from the viral strain F52/70 IBDV under a vaccinia promoter within the recombinant Fowlpox vaccine [10]. The whole of VP2 and part of VP4 were inserted in the same ORF as a β-galactosidase gene. This results in the production of a fusion protein, which allows for the identification of recombinant viruses. When administered at 1 and 14 days of age, fpIBD1 protected against mortality resulting from the challenge of 4-week-old RIR chickens with virulent and very virulent strains of IBDV, F52/70, and CS89, respectively [25].

No bursal damage in the Bursa of Fabricius was seen in RIR chickens that had been vaccinated with fpIBD1, and then challenged with IBDV strain F52/70 at doses of 10^1.3^ EID_50_ or 10^1.7^ EID_50_. The dose of challenge virus that breaks the protection stimulated by fpIBD1 was determined, and the virus challenge dose chosen for the challenge animal experiments was 10^2.3^ EID_50_ [36]. These results were obtained using outbred RIR chickens and are consistent with Shaw and Davison’s observations [13], by which protection mediated by fpIBD1 can be broken by increasing the dose of challenge virus. As at low challenge titers, fpIBD1 protected against bursal depletion as well as mortality, but at higher titers, bursal lesions were evident.

Interestingly, no antibody against IBDV was detectable after vaccination with fpIBD1 and prior to challenge with IBDV [10,13], which would suggest that only a cellular immune response is involved in the protective response and the control of IBDV, and that antibodies are not essential for providing protection. Also, IBDV does not infect T lymphocytes [37,38].

There are many possible views to explain why fpIBD1 does not provide complete protection. In the IBDV genome, VP2 is not expressed separately, but as part of a polyprotein that is cleaved by VP4 [39,40]. One possibility could be that only low levels of the fusion protein are produced by this recombinant. This could be due to the presence of a vaccinia promoter, or it could be that fpIBD1 may not express correctly folded VP2, due to the absence of VP4, or another possibility is that the expression of VP2 was at very low levels so did not stimulate an antibody response. A second possibility is that the fpIBD1 does not replicate very well in the chickens. However, antibodies against FPV were produced and detected [10,13], suggesting that the replication of fpIBD1 occurred. Finally, another possibility is that the VP2 antigen was not present in the correct conformation.

The Fowlpox virus genome contains many immunomodulatory genes, including an IL-18bp [12,20]. It seemed logical to delete this gene from vectors containing chIL-18 as an adjuvant. However, the Fowlpox virus presumably uses its vIL-18bp as part of a strategy to avoid the host immune response. Deleting the vIL-18bp gene might, therefore, have had an adverse effect on the persistence of the fpIBD1 vaccine in the host and could, therefore, have altered the magnitude of the immune response to the vaccine.

A recent study by Gibson et al. supports our conclusion that ORF214 in FPV is most likely to be IL-18bp. Their results demonstrated that ORF214 would be likely to inhibit IL-18 activity in the chicken by acting as IL-18bp, whereas ORF073 would not [41].

Our results showed that complete protection against IBDV can be provided after the insertion of chIL-18 into the recombinant fpIBD1, especially after knocking out the IL-18bp from the vector.

FPV presumably uses its vIL-18bp as part of a strategy to avoid the host immune response. ORF214 is the better candidate for the IL-18bp, as fpIBD1Δ214 gave better protection than fpIBD1 or fpIBD1Δ073. Despite the use of a challenge dose of IBDV, high enough to overcome protection and cause bursal damage in birds vaccinated with fpIBD1, there was no bursal damage in birds vaccinated with viruses containing chIL-18. Furthermore, no IBDV was detected in the bursae of birds vaccinated with fpIBD1Δ214::IL-18.

## 5. Conclusions

In conclusion, we believe our data indicate that chIL-18 can act as a vaccine adjuvant with fpIBD1 when challenged with a virulent strain of IBDV. Complete protection was provided against virulent IBDV challenge in chicken groups that had been vaccinated with altered fpIBD1 containing ChIL-18. In addition, based on the results of this study, we can conclude that ORF214 is a better candidate to be IL-18bp. Further studies are needed to confirm the efficacy of this vaccine against challenge with vvIBDV and VarIBDV strains. 

## Figures and Tables

**Table 1:** Titration of the six vaccines.

Virus	Final Concentration (pfu/mL)
fpIBD1	1 × 10^9^
fpIBD1Δ073	2 × 10^9^
fpIBD1Δ214	1 × 10^9^
fpIBD1::IL-18	2 × 10^8^
fpIBD1Δ073::IL-18	2 × 10^8^
fpIBD1Δ214::IL-18	3 × 10^8^

**Table 2:** Challenge experiment.

Room	Group	Treatment	No. of Birds
1	1	Unvaccinated and unchallenged (negative control)	3
2	2	Unvaccinated and challenged (positive control)	7
3	3	Vaccinated with fpIBD1 and challenged	7
	4	Vaccinated with fpIBD1Δ073 and challenged	7
	5	Vaccinated with fpIBD1Δ214 and challenged	7
	6	Vaccinated with fpIBD1::IL18 and challenged	7
	7	Vaccinated with fpIBD1Δ073::IL18 and challenged	7
	8	Vaccinated with fpIBD1Δ214::IL-18 and challenged	7
4	9	Vaccinated with fpIBD1	3
	10	Vaccinated with fpIBD1Δ073	3
	11	Vaccinated with fpIBD1Δ214	3
	12	Vaccinated with fpIBD1::IL18	3
	13	Vaccinated with fpIBD1Δ073::IL18	3
	14	Vaccinated with fpIBD1Δ214::IL-18	3

**Table 3:** Primers and probes for real-time quantitative RT-PCR.

RNA Target	Primer/Probe *	Sequence (5′-3′)
28S	F	GGC GAA GCC AGA GGA AAC T
	R	GAC GAC CGA TTT GCA CGT C
	Probe	AGG ACC GCT ACG GAC CTC CAC CA
IBDV (VP2)	F	GAG GTG GCC GAC CTC AAC T
	R	AGC CCG GAT TAT GTC TTT GAA G
	Probe	TCC CCT GAA GAT TGC AGG AGC ATT TG
IL-18/1	F	ATG AGC TGT GAA GAG ATC GC
IL-18/5	R	GGA CTG TTA CTG TAG CTG GC

* All probes were labelled with FAM (5′) and TAMRA (3′).

**Table 4:** Bursal damage scores for different groups after infection with vIBDV based on the histological scoring system of Muskett et al. [28].

Vaccination	IBDV Challenge	Bursal Damage Score *					
		0	1	2	3	4	5
-	-	5	-	-	-	-	-
-	+	-	-	-	1	3	1
fpIBD1	+	1	3	1	-	-	-
fpIBD1Δ073	+	2	2	1	-	-	-
fpIBD1Δ214	+	4	1	-	-	-	-
fpIBD1::IL-18	+	5	-	-	-	-	-
fpIBD1Δ073::IL-18	+	5	-	-	-	-	-
fpIBD1Δ214::IL-18	+	5	-	-	-	-	-

* Scoring system: 0 = No bursal damage in any follicle, clear demarcation of medulla and cortex. 1 = Mild necrosis of occasional follicles with overall bursal architecture maintained. 2 = <50% of follicles with severe lymphocyte depletion. 3 = >50% of follicles with severe lymphocyte depletion. 4 = Follicular outlines only remaining, increased connective tissue, cysts. 5 = Loss of all follicular architecture, fibroplasias.

## Data Availability

All data supporting the findings of this study are available within the manuscript. Any additional data are available from the corresponding author upon reasonable request.

## References

[1] Cosgrove A. (1962). An Apparently New Disease of Chickens: Avian Nephrosis. Avian Dis..

[2] Eterradossi N., Saif Y.M., Swayne D.E., Boulianne M., Logue C.M., McDougald L.R., Nair V., Suarez D.L., de Wit S., Grimes T., Johnson D., Kromm M. (2020). Infectious bursal disease. Diseases of Poultry.

[3] Gao H., Wang Y., Gao L., Zheng S.J. (2023). Genetic Insight into the Interaction of IBDV with Host—A Clue to the Development of Novel IBDV Vaccines. Int. J. Mol. Sci..

[4] Van der Berg T.P., Gonze M., Meulemans G. (1991). Acute infectious bursal disease in poultry: Isolation and characterisation of a highly virulent strain. Avian Pathol..

[5] Hein R., Koopman R., García M., Armour N., Dunn J.R., Barbosa T., Martinez A. (2021). Review of Poultry Recombinant Vector Vaccines. Avian Dis..

[6] Taylor J., Edbauer C., Rey-Senelonge A., Bouquet J.F., Norton E., Goebel S., Desmettre P., Paoletti E. (1990). Newcastle disease virus fusion protein expressed in a fowlpox virus recombinant confers protection in chickens. J. Virol..

[7] Criado M.F., Bertran K., Lee D.-H., Killmaster L., Stephens C.B., Spackman E., e Silva M.S., Atkins E., Mebatsion T., Widener J. (2019). Efficacy of novel recombinant fowlpox vaccine against recent Mexican H7N3 highly pathogenic avian influenza virus. Vaccine.

[8] Qingzhong Y., Barrett T., Brown T.K., Cook J.K., Green P., Skinner M.A., Cavanagh D. (1994). Protection against turkey rhinotracheitis pneumovirus (TRTV) induced by a fowlpox virus recombinant expressing the TRTV fusion glycoprotein (F). Vaccine.

[9] Lee L.F., Bacon L.D., Yoshida S., Yanagida N., Zhang H.M., Witter R.L. (2004). The Efficacy of Recombinant Fowlpox Vaccine Protection Against Marek’s Disease: Its Dependence on Chicken Line and B Haplotype. Avian Dis..

[10] Bayliss C.D., Peters R.W., Cook J.K.A., Reece R.L., Howes K., Binns M.M., Boursnell M.E.G. (1991). A recombinant fowlpox virus that expresses the VP2 antigen of infectious bursal disease virus induces protection against mortality caused by the virus. Arch. Virol..

[11] Fahey K.J., Erny K., Crooks J. (1989). A conformational immunogen on VP2 of infectious bursal disease virus that induce virus-neutralizing antibodies that passively protect chickens. J. Gen. Virol..

[12] Laidlaw S.M., Skinner M.A. (2004). Comparison of the genome sequence of FP9, an attenuated, tissue culture-adapted European strain of Fowlpox virus, with those of those virulent American and European viruses. J. Gen. Virol..

[13] Shaw I., Davison T. (2000). Protection from IBDV-induced bursal damage by a recombinant fowlpox vaccine, fpIBD1, is dependent on the titre of challenge virus and chicken genotype. Vaccine.

[14] Schneider K., Puehler F., Baeuerle D., Elvers S., Staeheli P., Kaspers B., Weining K.C. (2000). cDNA cloning of biologically active chicken interleukin-18. J. Interferon Cyt. Res..

[15] Hung L.-H., Li H.-P., Lien Y.-Y., Wu M.-L., Chaung H.-C. (2010). Adjuvant effects of chicken interleukin-18 in avian Newcastle disease vaccine. Vaccine.

[16] Chen H.-Y., Yang M.-F., Cui B.-A., Cui P., Sheng M., Chen G., Wang S.-J., Geng J.-W. (2010). Construction and immunogenicity of a recombinant fowlpox vaccine coexpressing S1 glycoprotein of infectious bronchitis virus and chicken IL-18. Vaccine.

[17] Chen H.-Y., Shang Y.-H., Yao H.-X., Cui B.-A., Zhang H.-Y., Wang Z.-X., Wang Y.-D., Chao A.-J., Duan T.-Y. (2011). Immune responses of chickens inoculated with a recombinant fowlpox vaccine coexpressing HA of H9N2 avain influenza virus and chicken IL-18. Antivir. Res..

[18] Chen H.Y., Cui P., Cui B.A., Li H.P., Jiao X.Q., Zheng L.L., Cheng G., Chao A.J. (2011). Immune responses of chickens inoculated with a recombinant fowlpox vaccine coexpressing glycoprotein B of infectious laryngotracheitis virus and chicken IL-18. FEMS Immunol. Med. Microbiol..

[19] Shi W., Liu Q., Zhang J., Sun J., Jiang X., Geng J., Wang F., Xiao Y., Li H., Zhao X. (2014). Co-expression of EtMic2 protein and chicken interleukin-18 for DNA vaccine against chicken coccidiosis. Res. Vet. Sci..

[20] Afonso C.L., Tulman E.R., Lu Z., Zsak L., Kutish G.F., Rock D.L. (2000). The Genome of Fowlpox Virus. J. Virol..

[21] Laidlaw S.M., Anwar M.A., Thomas W., Green P., Shaw K., Skinner M.A. (1998). Fowlpox Virus Encodes Nonessential Homologs of Cellular Alpha-SNAP, PC-1, and an Orphan Human Homolog of a Secreted Nematode Protein. J. Virol..

[22] Nooruzzaman M., Hossain I., Rahman M.M., Uddin A.J., Mustari A., Parvin R., Chowdhury E.H., Islam M.R. (2022). Comparative pathogenicity of infectious bursal disease viruses of three different genotypes. Microb. Pathog..

[23] Trapp J., Rautenschlein S. (2022). Infectious bursal disease virus’ interferences with host immune cells: What do we know?. Avian Pathol..

[24] Wyatt L.S., Earl P.L., Moss B. (2017). Generation of Recombinant Vaccinia Viruses. Curr. Protoc. Protein Sci..

[25] Rothwell C., Vervelde L., Davison T. (1996). Identification of chicken Bu-1 alloantigens using the monoclonal antibody AV20. Vet. Immunol. Immunopathol..

[26] Techera C., Tomás G., Panzera Y., Banda A., Perbolianachis P., Pérez R., Marandino A. (2018). Development of real-time PCR assays for single and simultaneous detection of infectious bursal disease virus and chicken anemia virus. Mol. Cell. Probes.

[27] Bayliss C.D., Spies U., Shaw K., Peters R.W., Papageorgiou A., Muller H., Boursnell M.E.G. (1990). A comparison of the sequences of segment A of four infectious bursal disease virus strains and identification of a variable region in VP2. J. Gen. Virol..

[28] Muskett J., Hopkins I., Edwards K., Thornton D. (1979). Comparison of two infectious bursal disease vaccine strains: Efficacy and potential hazards in susceptible and maternally immune birds. Veter. Rec..

[29] Degen W.G., van Daal N., Rothwell L., Kaiser P., Schijns V.E. (2005). Th1/Th2 polarization by viral and helminth infection in birds. Veter Microbiol..

[30] Eldaghayes I., Rothwell L., Williams A., Withers D.R., Balu S., Davison F., Kaiser P., Hu T., Wu Z., Vervelde L. (2006). Infectious Bursal Disease Virus: Strains That Differ in Virulence Differentially Modulate the Innate Immune Response to Infection in the Chicken Bursa. Viral Immunol..

[31] Gelb J., Eidson C.S., Fletcher O.J., Kleven S.H. (1979). Studies on Interferon Induction by Infectious Bursal Disease Virus (IBDV). II. Interferon Production in White Leghorn Chickens Infected with an Attenuated or Pathogenic Isolant of IBDV. Avian Dis..

[32] Rautenschlein S., Yeh H.-Y., Sharma J.M. (2003). Comparative Immunopathogenesis of Mild, Intermediate, and Virulent Strains of Classic Infectious Bursal Disease Virus. Avian Dis..

[33] Bagheri S., Paudel S., Wijewardana V., Kangethe R.T., Cattoli G., Hess M., Liebhart D., Mitra T. (2022). Production of interferon gamma and interleukin 17A in chicken T-cell subpopulations hallmarks the stimulation with live, irradiated and killed avian pathogenic Escherichia coli. Dev. Comp. Immunol..

[34] Rautenschlein S., Alkie T.N. (2016). Infectious bursal disease virus in poultry: Current status and future prospects. Vet. Med. Res. Rep..

[35] Dey S., Pathak D.C., Ramamurthy N., Maity H.K., Chellappa M.M. (2019). Infectious bursal disease virus in chickens: Prevalence, impact, and management strategies. Vet. Med. Res. Rep..

[36] Haygreen E., Kaiser P., Burgess S., Davison T. (2006). In ovo DNA immunisation followed by a recombinant fowlpox boost is fully protective to challenge with virulent IBDV. Vaccine.

[37] Vervelde L., Davison T.F. (1997). Comparison of the *in situ* changes in lymphoid cells during infection with infectious bursal disease virus in chickens of different ages. Avian Pathol..

[38] Dai M., Xu C., Chen W., Liao M. (2019). Progress on chicken T cell immunity to viruses. Cell. Mol. Life Sci..

[39] Heine H.-G., Boyle D.B. (1993). Infectious bursal disease virus structural protein VP 2 expressed by a fowlpox virus recombinant confers protection against disease in chickens. Arch. Virol..

[40] Lee M., Doong S., Lai S., Ho J., Wang M. (2006). Processing of Infectious Bursal Disease Virus (IBDV) Polyprotein and Self-Assembly of IBDV-Like Particles in Hi-5 Cells. Biotechnol. Prog..

[41] Gibson M.S., Steyn A., Kealy D., Kaspers B., Fife M.S. (2020). Molecular cloning and characterisation of chicken IL-18 binding protein. Dev. Comp. Immunol..

